# What are the important surgical factors affecting the wound healing after primary total knee arthroplasty?

**DOI:** 10.1186/s13018-016-0340-y

**Published:** 2016-01-13

**Authors:** Kengo Harato, Hidenori Tanikawa, Yutaro Morishige, Kazuya Kaneda, Yasuo Niki

**Affiliations:** Department of Orthopedic Surgery, Keio University School of Medicine, 35 Shinanomachi, Shinjuku-ku, Tokyo, 160-8582 Japan; Department of Orthopedic Surgery, Kawasaki Municipal Kawasaki Hospital, 12-1 Shinkawadouri, Kawasakiku, Kawasaki City, Kanagawa prefecture 210-0013 Japan

## Abstract

**Background:**

Wound condition after primary total knee arthroplasty (TKA) is an important issue to avoid any postoperative adverse events. Our purpose was to investigate and to clarify the important surgical factors affecting wound score after TKA.

**Methods:**

A total of 139 knees in 128 patients (mean 73 years) without severe comorbidity were enrolled in the present study. All primary unilateral or bilateral TKAs were done using the same skin incision line, measured resection technique, and wound closure technique using unidirectional barbed suture. In terms of the wound healing, Hollander Wound Evaluation Score (HWES) was assessed on postoperative day 14. We performed multiple regression analysis using stepwise method to identify the factors affecting HWES. Variables considered in the analysis were age, sex, body mass index (kg/m^2^), HbA1C (%), femorotibial angle (degrees) on plain radiographs, intraoperative patella eversion during the cutting phase of the femur and the tibia in knee flexion, intraoperative anterior translation of the tibia, patella resurfacing, surgical time (min), tourniquet time (min), length of skin incision (cm), postoperative drainage (ml), patellar height on postoperative lateral radiographs, and HWES. HWES was treated as a dependent variable, and others were as independent variables.

**Results:**

The average HWES was 5.0 ± 0.8 point. According to stepwise forward regression test, patella eversion during the cutting phase of the femur and the tibia in knee flexion and anterior translation of the tibia were entered in this model, while other factors were not entered. Standardized partial regression coefficient was as follows: 0.57 in anterior translation of the tibia and 0.38 in patella eversion.

**Conclusions:**

Fortunately, in the present study using the unidirectional barbed suture, major wound healing problem did not occur. As to the surgical technique, intraoperative patella eversion and anterior translation of the tibia should be avoided for quality cosmesis in primary TKA.

## Background

Wound condition after primary total knee arthroplasty (TKA) is important for the prevention of periprosthetic infection [1, 2]. Any delay in wound healing will cause deep infection, which leads to the arthroplasty failure. For example, in a retrospective review of 17,000 TKAs described by Galat et al. [3], the consequences of the early wound complications were of great significance as the probability of further major surgery operations (removal of implants, muscle flap rotation, leg amputation) or diagnosis of deep infection were 5.3 and 6.0 %, respectively, within 2 years of primary procedure. On the other hand, TKAs at 2 years without postoperative wound complications required a major operation or were diagnosed with a deep infection of 0.6 and 0.8 %, respectively. Prevention of soft tissue problems is thus essential to achieve excellent clinical results. According to previous reports, important considerations affecting the surgical wound healing after TKA were the proper selection of skin incision, an understanding of vascular anatomy around the knee, patient risk factors, appropriate postoperative care, and prompt management [1, 2, 4]. For instance, patient risk factors include prior open surgical procedures at the same site, immunosuppressive therapy, hypokalemia, poor nutrition (albumin <3.4 g/dl), diverticulosis, infection elsewhere, poorly controlled diabetes mellitus, obesity, smoking, renal failure, hypothyroidism, and alcohol abuse [2, 5–9]. Surgical factors include the location of the incision, poor soft tissue handling, and longer tourniquet use. Postoperative risk factors for wound complications include tight dressings, large subcutaneous hematomas, very aggressive physiotherapy, and accelerated continuous passive motion [1, 2, 10, 11]. However, when patients without any risk factors undergo the primary TKA, it is unknown as to the important surgical factors affecting the wound healing using detailed wound score after primary TKA so far.

It was hypothesized that operative technique would affect wound healing in primary TKA. The purpose of the present study was to investigate and to clarify the important surgical factors affecting wound score after primary TKA.

## Methods

### Subjects

A total of 139 knees in 128 patients (mean 73 years) with end-stage knee osteoarthritis (121 knees) or osteonecrosis (18 knees) were enrolled. Strict inclusion criteria were established in the present study. Patients with immunosuppressive therapy, hypokalemia, poor nutrition (albumin <3.4 g/dl), diverticulosis, infection elsewhere, uncontrolled diabetes mellitus (HbA1C >7.0 %), obesity (body mass index >35 kg/m^2^), smoking, renal failure, hypothyroidism, alcohol abuse, rheumatoid arthritis, posttraumatic arthritis, or previous knee surgery were excluded. All the patients underwent primary TKA by the same surgeon (K.H.). They underwent unilateral or bilateral TKA using Balanced Knee System®, posterior stabilized (PS) design (Ortho Development, Draper, UT), or Legion®, PS design (Smith and Nephew, Memphis, TN) under general and/or epidural anesthesia. General and epidural anesthesia were selected for most of the patients. General anesthesia without the epidural anesthesia was selected for patients who underwent the previous spinal surgery.

### Surgical technique

The operative technique consisted of medial parapatellar skin incision, less invasive medial midvastus approach, intramedullary rod in the femoral cuts, extramedullary guide in the tibial cuts, and spacer block to make appropriate ligament balance. Patella resurfacing was selected depending on the damage to the cartilage during the procedure. In patella resurfacing, patella eversion was done in the extended position of the knee joint. Patella eversion during the cutting phase of the femur and tibia in knee flexion was done according to surgeon’s preference. In fact, patella eversion during the cutting phase of the femur and tibia was done in 72 cases (the first half) and not done in 67 cases (the latter half). The infrapatellar fat pad was resected in all patients. All the permanent components were fixed with use of bone cement. With respect to bone resection and component fixation of the tibia, anterior translation was selected in Legion® (36 knees) despite the fact that it was not necessary for Balanced Knee System® (103 knees) because the component design of the tibia, especially in the length of the keel, is different between these designs. Concerning the closure, capsule was repaired using interrupted number 1 braided absorbable sutures and 1-0 unidirectional barbed suture (V-Loc, Covidien, Mansfield, MA). Thereafter, closure of subdermal layer was done using a 3-0 monofilament absorbable suture with inverted interrupted knots and 4-0 unidirectional barbed suture (V-Loc, Covidien, Mansfield, MA). Final skin closure was done with adhesive tape, and an elastic compression dressing (Aquacel Surgical cover dressing, ConvaTec, NJ) was applied. Patients underwent a standard rehabilitation program consisting of early range of motion and weight-bearing as tolerated. Edoxaban 15 mg was used from postoperative day 2 to 14 for the prophylaxis of deep vein thrombosis in all patients based on our standard protocol. Drains were removed on postoperative day 2. Patients underwent a standard rehabilitation program which consisted of early range of motion and weight-bearing exercises as tolerated.

### Evaluations

A retrospective observational study was conducted at our institution. All the subjects provided informed consent and the study was approved by our institution (no. 20150043). In terms of wound healing, Hollander Wound Evaluation Score (HWES) was assessed on postoperative day 14. HWES is a validated and widely used cosmetic scoring system [12]. This is consisted of a cosmetic score from 0 (worst result) to 6 (best result) based on the presence or absence of six features, namely, step-off of borders, contour irregularities, margin separation, edge inversion, excessive distortion, and overall appearance (Table 1). In addition, wound-related adverse events, including superficial infection, deep infection wound dehiscence, and suture abscess, were assessed in all cases on postoperative day 21.

**Table 1:** Hollander Wound Evaluation Score (HWES) [12]

Hollander Wound Evaluation Score (HWES)
1. Step-off borders
2. Contour irregularity—puckering
3. Scar width—greater than 2 mm
4. Edge inversion—sinking, curling
5. Inflammation—redness, discharge
6. Overall cosmesis
6/6 optional wound healing

We evaluated age, sex, body mass index (BMI), HbA1C (%), preoperative femorotibial (FTA) angle on plain radiograph, and patellar height on postoperative lateral radiographs. FTA was defined as the lateral angle formed by the femur and tibia. Lines were drawn through the middle of the femoral shaft and through the middle of the tibial shaft. Patellar height was calculated using Insall-Salvati ratio on postoperative lateral radiographs. In addition, intraoperative patella eversion during the cutting phase in knee flexion, intraoperative anterior translation of the tibia, patella resurfacing, surgical time, tourniquet time, length of skin incision, the amount of postoperative drainage were also evaluated as surgical factors.

### Statistical analysis

We performed multiple regression analysis using stepwise method to identify the factors affecting HWES. Variables considered in the analysis were age, sex, BMI, HbA1C, FTA, intraoperative patella eversion, intraoperative anterior translation of the tibia, patella resurfacing, surgical time, tourniquet time, length of skin incision, patellar height, and HWES. HWES was treated as a dependent variable, and others were as independent variables. All statistical analyses were done with the use of SPSS® Version 22 for Microsoft Windows (Chicago, IL). *P* values of <0.05 were considered as significant.

## Results

Detailed demographic data was presented in Table 2. The average HWES was 5.0 ± 0.8 point. As to the distribution of the wound score, HWES was 3 points in 5 cases, 4 in 29 cases, 5 in 63 cases, and 6 in 42 cases. As any adverse events were not found in each TKA, there were no cases requiring debridement and re-closure of the wound after primary TKA. Basically, unidirectional barbed suture provided the good results in HWES.

**Table 2:** Detailed data of all operations (mean ± SD)

Evaluations (*N* = 139)	
Age (years)	72.9 ± 8.1
Sex (female/male)	108/31
BMI (kg/m^2^)	25.8 ± 3.9
HbA1C (%)	5.8 ± 0.5
FTA (deg.)	183.7 ± 8.4
Patella eversion (+/−)	72/67
Tibial translation (+/−)	36/103
Surgical time (min)	105.9 ± 13.2
Tourniquet time (min)	73.5 ± 16.8
Patella resurfacing (+/−)	105/34
Length of skin incision (cm)	12.7 ± 1.5
Postoperative drainage (ml)	479 ± 168
Patellar height	1.26 ± 0.13
HWES	5.0 ± 0.8

According to stepwise forward regression test, intraoperative patella eversion and anterior translation of the tibia were entered in this model, while age, Sex, BMI, HbA1C, FTA, surgical time, tourniquet time, patella resurfacing, length of skin incision, and patellar height were not entered in the model (Table 3). The multiple regression analysis in the current study would show the appropriate results, as Durbin-Watson ratio was 1.762. Standardized partial regression coefficient was as follows: 0.57 in anterior translation of the tibia and 0.38 in patella eversion (Table 3).

**Table 3:** Detailed information of the multiple regression analysis using stepwise method

	Partial regression coefficient	Standardized partial regression coefficient	*P*	95 % confidence interval
Patella eversion	0.57	0.35	0.001>	0.31–0.82
Tibial translation	0.38	0.21	0.009	0.10–0.67

*R* ^2^ = 0.15, ANOVA *P* < 0.0001; Durbin-Watson ratio 1.762

## Discussion

The results of the present study would support out hypothesis that operative technique would affect wound healing in primary TKA. Specifically, patella eversion and anterior translation of the tibia were related to low score of the wound healing. TKA without patella eversion and anterior translation of the tibia is well known as the minimally invasive technique. Proponents of the minimally invasive technique claim that these techniques will result in an earlier recovery of range of motion, an earlier discharge from the hospital, and less pain compared with the conventional TKA without any complications including the wound problems [13]. Dalury et al. indicated that patellar eversion and anterior tibial translation could have no adverse effects on the range of motion, quadriceps strength, or patient’s knee preference during the early postoperative recovery period after TKA [14]. However, their study did not investigate the wound healing problem.

In the present investigation, inclusion criteria were strictly established and postoperative rehabilitation was done using the same protocol at our institution. Therefore, only surgical factors could be assessed and discussed. Several surgical factors affecting the wound healing after TKA has been reported so far. For example, surgical factors include the location of the incision, longer tourniquet use, and poor soft tissue handling. Previous studies indicated that medial parapatellar skin incision provided the worse outcome of the wound healing than anterior midline skin incision [10]. Nevertheless, in the more recent study, wound healing of these two incisions were reported to be similar [15]. In the current study, for that reason, medial parapatellar skin incision was used. Concerning the tourniquet time, Olivecrona et al. reported that tourniquet time over 100 min was associated with an increased risk of complications after TKA (OR 2.2, CI 1.5–3.1) [11]. In our study, all procedures were done by the same surgeon and using a tourniquet for an average of 73 min, which seemed not to influence on the wound evaluation score. As to the soft tissue handling, previous reports suggested that the distal part of the skin incision was significantly more hypoxic than the proximal part in TKA [16, 17]. Therefore, atraumatic wound edge retraction should be carried out especially in the distal part. However, except for this technique, gentle handling of the soft tissue has not been investigated in detail. Reification of the technique has not been made in the handling methods. Detailed surgical technique affecting the wound healing was thus still unknown. From the present study, intraoperative patella eversion and anterior translation of the tibia during the cutting phase in knee flexion would lead to wound hypoxia of the edge, and thus, those should be avoided for wound healing in primary TKA.

Recently, barbed suture has been used for closure of TKA [18–22]. Actually, safety and efficacy of barbed suture have been reported in several studies. However, especially in TKA, previous studies indicated that the use of the barbed suture would lead to higher complication rate especially in wound healing compared with conventional staples [4, 23]. Potentially, the tightness and water-tight seal provided by the barbed suture are less forgiving than those by a conventional suture to the high stresses of postoperative mobilization and normal physiologic drainage after TKA [4]. On the other hand, it is possible that barbed suture may lead to quality cosmesis and better wound score in some cases [23]. As to the HWES, average value was similar to the recent study using the barbed suture [24]. Fortunately, major wound problems did not occur in the present study, although unidirectional barbed suture was used in all cases. Contrary to previous reports, patients with uncontrolled diabetes mellitus, smoker, obesity, and any severe comorbidity were excluded, and wound drainage was performed for 2 days postoperatively to decrease knee effusion. Moreover, wound condition was carefully assessed for 2-week admission in the present study. Presumably, for that reason, major wound healing problems did not occur.

Several limitations should be noted in the present study. First, the weakness of the present study was that this was not performed as a prospective randomized trial study. Second, the current study was done and assessed by a single surgeon at our institution. Thus, our study may not be representative of the results obtained by other surgeons in other countries. Third, serum albumin level was assessed preoperatively, though wound healing could be affected by postoperative nutrition condition. Fourth, the use of one design was optimal for the material. Actually, surgical techniques were different between the two designs because the component design of the tibia, especially in length of the keel, is different between these designs. Lastly, as the healing of the surgical wound was basically acceptable regardless of wound score in the present study, it was less meaningful except for those who would like to obtain quality cosmesis of the surgical wound in TKA. However, as the current study was the first to examine the important surgical factors affecting wound score after primary TKA, our results offer useful information that patella eversion and anterior translation of the tibia were related to low score of the wound healing following TKA.

## Conclusions

Fortunately, in the present study using the unidirectional barbed suture, major wound healing problem did not occur. As to the surgical technique, intraoperative patella eversion and anterior translation of the tibia during the cutting phase in knee flexion should be avoided for quality cosmesis of the wound healing in primary TKA.
